# Kentucky Bluegrass Invasion in the Northern Great Plains and Prospective Management Approaches to Mitigate Its Spread

**DOI:** 10.3390/plants10040817

**Published:** 2021-04-20

**Authors:** Rakhi Palit, Greta Gramig, Edward S. DeKeyser

**Affiliations:** 1 School of Natural Resource Sciences, North Dakota State University, Fargo, ND 58102, USA; edward.dekeyser@ndsu.edu; 2 Department of Plant Sciences, North Dakota State University, Fargo, ND 58102, USA; Greta.Gramig@ndsu.edu

**Keywords:** climate change, competitiveness, ecosystem, fire, grazing, invasion, interactions, Kentucky bluegrass, management, native species, Northern Great Plains

## Abstract

Kentucky bluegrass (*Poa pratensis* L.) is one of the most aggressive grasses invading Northern Great Plains (NGP) grasslands, resulting in substantial native species losses. Highly diverse grasslands dominated by native species are gradually transforming into rangelands largely dominated by non-native Kentucky bluegrass. Several factors potentially associated with Kentucky bluegrass invasions, including high propagule pressure, thatch formation, climate change, and increasing nitrogen deposition, could determine the future dominance and spread of Kentucky bluegrass in the NGP. Because atmospheric CO_2_ is amplifying rapidly, a C3 grass like Kentucky bluegrass might be photosynthetically more efficient than native C4 grasses. As this exotic species shares similar morphological and phenological traits with many native cool-season grasses, controlling it with traditional management practices such as prescribed fire, grazing, herbicides, or combinations of these practices may also impair the growth of native species. Thus, developing effective management practices to combat Kentucky bluegrass spread while facilitating the native species cover is essential. Modifying traditional techniques and embracing science-based adaptive management tools that focus on the ecological interactions of Kentucky bluegrass with the surrounding native species could achieve these desired management goals. Enhancement of the competitiveness of surrounding native species could also be an important consideration for controlling this invasive species.

## 1. Introduction

Grasslands are one of the most endangered ecosystems in North America [1,2,3,4,5]. The rapid spread of invasive plant species caused many grassland ecosystems to be dominated by invasive plants [6,7,8]. The threat posed by the invasion of non-native species is considered to be the second most significant factor contributing to the endangerment of native species after land clearing and habitat fragmentation [9,10]. Throughout the Great Plains, approximately 70% of grasslands have been lost, leaving only about 13% of the original extent of tallgrass, 29% of mixed-grass, and 52% of shortgrass prairies [1,11]. This is primarily due to agricultural conversions that have transformed the NGP into the most threatened yet least protected ecosystem [1,12,13]. For example, in the NGP, North Dakota and South Dakota retain less than 3% of the original tallgrass prairies [11,14]. To protect these ecosystems and preserve biodiversity, conservation and restoration of the remnant prairies throughout the NGP are extremely important [5,11,14].

Invasions of non-native species, or biological invasions, cause significant ecological and economic impacts worldwide, affecting both managed and native ecosystems [15] as well as human health [16,17]. With the increasing number of invasive species, the risks associated with non-native plants are becoming widely recognized [18,19]. Kentucky bluegrass (*Poa pratensis* L.) is one of the most aggressive non-native grasses spreading across the Great Plains [6,11,12,13]. This grass is mainly used as a turfgrass in the Great Plains, especially for lawns and golf courses [3,20,21]. Kentucky bluegrass grass also has been historically used for pasture [3,22]. This species can be used as a nutritious forage during certain periods of the year, except the summer, and it also has been useful as a soil stabilizer to control erosion [6,23,24]. In the NGP, Kentucky bluegrass invasion is associated with a considerable decline in native plant diversity and changes in community structure and function [6,25,26]. Thus, invasion by this species could potentially result in the loss of many critical ecological services through trophic downgrading effects [6,27]. Ongoing climate change and increasing nitrogen deposition also promote invasive rangeland species such as Kentucky bluegrass by suppressing the growth of native species. [3,6]. Dislocation and replacement of native species by specific invasive species often transform a highly diverse ecosystem into a homogenized emerging or novel ecosystem [28,29]. Traditional management practices that are used to control Kentucky bluegrass include prescribed fire, grazing, application of herbicides, and a combination of these tactics [19,29,30,31,32]. As Kentucky bluegrass morphological and physiological traits resemble those of many surrounding native cool-season grasses, controlling this species with traditional management practices also can impair the growth and spread of those native species [6,30].

In this review article, we discuss the potential factors that might regulate the future dominance of Kentucky bluegrass in the Northern Great Plains and the challenges associated with the management of this invasive species. Our goal is to discuss prospective science-based adaptive management practices that would simultaneously combat Kentucky bluegrass cover while promoting the native species cover. We provide a systematic review of the studies investigating Kentucky bluegrass invasion in the NGP and its management practices. Moreover, we review how climate change and increased nitrogen deposition are promoting invasive species cover in rangelands and how amendments in traditional practices and incorporation of ecological research would aid the development of effective management strategies for Kentucky bluegrass.

## 2. Distribution and Ecophysiology of Kentucky Bluegrass

Kentucky bluegrass accounts for about 39% of exotic species cover [6,26], spreading dramatically throughout the prairies in North Dakota and South Dakota [1,3]. Reinforcing this, analysis of National Resources Inventory rangeland data [33] revealed that Kentucky bluegrass occupies more than 50% of the sampled lands in North Dakota [6]. Kentucky bluegrass was likely introduced to North America during the 1600s [34,35], introduced to eastern North Dakota around 1890, and now comprises over 14% of the foliar canopy in the rangelands of this region [3,30]. In North Dakota, Kentucky bluegrass cover has increased to as much as 86% between 2011 and 2015 on some rangelands [30]. Northern latitudes and more temperate climates of Eurasia comprise the native range of Kentucky bluegrass [3,36]. This non-native, grazing-tolerant, cool-season, perennial C3 grass commonly grows in moist, well-drained soils [6,20,37]. Under favorable climatic conditions, its strong rhizomatous sod-forming growth habit makes this species a successful invader [3,6,36]. This grass forms dense “thatch,” or a firmly combined matrix of root, stem, and leaves forming cover soil surfaces, which functions as a growth substrate for roots and lateral stems [38]. Rapid propagation of Kentucky bluegrass occurs both by rhizomes and seed production [20,22]. Stem branching via fast tillering also results in the prolific biomass production associated with this species [22]. Compared to North American native grasses, Kentucky bluegrass seeds remain viable for an extended period. For example, Garrison and Stier [39] reported that after 22 months, this species exhibited between 12% and 24% seed viability, whereas native grasses such as *Andropogon gerardii, Panicum virgatum,* and *Elymus virginicus* retained only 0–1% of their viability [20]. Similarly, Setter and Lym (2013) [40] reported about 250% increase of Kentucky bluegrass in the seedbank in western North Dakota over ten years following successful leafy spurge (*Euphorbia esula*) biocontrol, a rate of increase much greater than other native grasses and forbs found in that area [3].

## 3. Multifactorial Impacts on Ecosystem Stability and Functioning

Invasions of non-native species profoundly affect ecosystem functioning and integrity [6,41,42], because some invaders can transform the way ecosystems function [43,44]. Such invaders can alter key ecosystem processes, such as nutrient cycling and fire frequency, and once initiated, these changes can be reinforced by feedbacks that make them difficult or impossible to reverse. For example, following nitrogen fertilization or soil disturbance, Kentucky bluegrass can potentially dislocate little bluestem (*Schizachyrium scoparium*), an important native, perennial, C4 grass in North American tallgrass prairies [6,45,46]. In a plant community where Kentucky bluegrass emerges as the dominant species, area cover and diversity of native grasses and forbs decrease dramatically [4,25,47]. DeKeyser et al. (2009) [4] revealed that over 23 years (1984–2007) at the Knife River Indian Villages National Historic Site in North Dakota, Kentucky bluegrass increased from 4 to 22%, while native forb species decreased from 34 to 14% on sandy soil; whereas on loamy soil, when Kentucky bluegrass cover increased from 13 to 34%, native grasses and forbs cover reduced from 66 to 4% and 24 to 12%, respectively. These findings showed that when Kentucky bluegrass becomes dominant, it can affect the surrounding environment and limit niches for other subdominant plants [6,48].

Plant–soil feedbacks can partially explain how exotic plant species invade and subsequently alter ecosystem functioning [49,50,51,52]. Plant–soil feedbacks arise because plant species differentially alter soil communities by cultivating a microbiota specific to each species. Soil microbial communities, including mycorrhizae and soil pathogens, can exert a strong influence on plant performance [53], abundance, and community structure [51], and this could be particularly important for invasive plants. For example, a previous study reported that soil fungi enhanced the competitive ability of an exotic weed species, *Centaurea maculosa*, more than the native grass *Festuca idahoensis* [54]. When native soil-borne pathogens accumulate in the rhizosphere of both exotic and native species, the native pathogens might provide a greater competitive advantage to the exotic species over the native competitors [49,55]. On the other hand, an exotic species might simply perform better in a new range because it escaped pathogens in its previous range, according to the enemy release hypothesis [55]. After establishing in the invaded range, Kentucky bluegrass can potentially alter plant–soil feedback mechanisms associated with native species. For instance, changes in the litter–soil-nutrient dynamics provide a competitive advantage to this species and ensure its dominance by dislocating the other native species in the community [49,50]. In general, invasive C3 grasses contain greater nitrogen concentrations and lower recalcitrant carbon than some native C4 grasses, which can cause increasing decomposition rates and faster nutrient cycling [51,52]. Greater plant and litter production by invasive C3 grasses might also enhance the soil microbial activity and soil nitrogen mineralization processes [51,53]. A shift to a Kentucky bluegrass-dominated prairie from the mixed-grassland can also have multidimensional effects on many organisms residing at different trophic levels, from underground dwellers to the organisms that reside on the soil surface [6]. Finally, similar to other sod-forming grasses, Kentucky bluegrass can also alter soil hydrology by reducing water infiltration into the soil and increasing the surface water runoff [51,54]. Thus, Kentucky bluegrass can function as a ”transformer” species as it not only affects the native plant community composition, but also soil nutrient cycling and other ecosystem processes with subsequent impacts on macro-and micro-organisms living in the prairies.

## 4. Drivers of the Successful Invasion of Kentucky Bluegrass

### 4.1. Propagule Pressure

**Figure 1:**
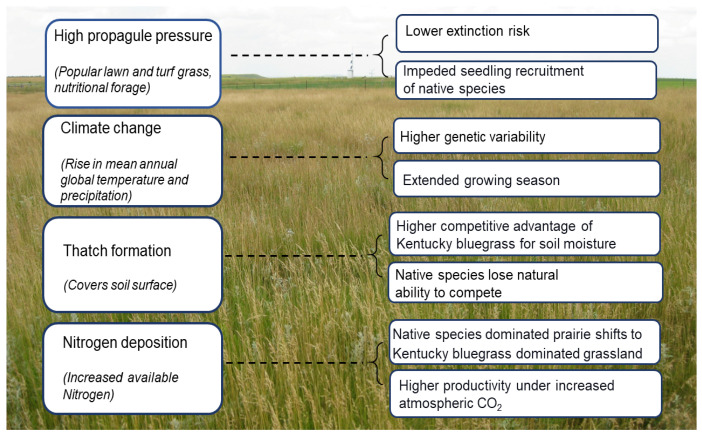
Factors influencing invasion and spread of Kentucky bluegrass in NGP. The background picture shows monocultural Kentucky bluegrass cover in native mixed-grass prairie in North Dakota. Background photo was taken by Dave Dewald.

Propagule pressure is the composite number of individual propagules of a non-native species introduced to a new region in single or multiple events [44,55] (Figure 1). Previous studies have reported a strong positive relationship between propagule pressure and successful naturalization and spread [56,57,58]. The release of many propagules enables the introduced species to overcome the risk of extinction associated with small populations [59] and for propagules to arrive at a ”safe site” [60]. High propagule pressure may also improve the chances of establishment by increasing the amount of genetic variation in the introduced population, reducing potential genetic bottlenecks and improving the chances of adapting successfully to new selection pressures in the recipient location [55,58]. Because Kentucky bluegrass is widely used as a lawn grass and turfgrass of the NGP, propagules are plentiful [3,61]. Approximately 250 Kentucky bluegrass cultivars are grown in the United States [62], and this species serves as the dominant component of many turfgrass industry [61]. Besides, this species is also popular as a nutritious livestock forage. Thus, with the steady supply of propagules resulting from widespread use, Kentucky bluegrass has a strong likelihood of escaping from cultivation to the wild in the NGP. However, recent genetic studies of wild populations of Kentucky bluegrass in this region have indicated propagule pressure might not play as critical a role compared to changes in land use and climate [63].

### 4.2. Thatch Formation

Kentucky bluegrass forms heavy thatch on the soil surface; this thatch and associated plant litter hinder the seedling recruitment of other native grasses and forbs [64]. Thatch has a lower water-holding capacity than soil, and it dries out rapidly, thus reduces the seed to soil contact needed for successful germination of many plant species. Because of the abundance of Kentucky bluegrass roots in the soil, this species also gains a competitive advantage for obtaining soil moisture [64]. Thus, the formation of thatch promotes Kentucky bluegrass spread while suppressing native species establishment.

### 4.3. Climate Change

Climate change may also affect Kentucky bluegrass invasions in the NGP [3]. Over the last 120 years, the growing season in North Dakota has extended by 12 days [65]. This would favor the growth and spread of Kentucky bluegrass due to earlier springs and later falls as it would be photosynthetically active during those times [3]. As Kentucky bluegrass produces more rhizomes in the fall, an extended fall would increase its vegetative reproductive capacity (Figure 1). In addition, earlier growth in the spring compared to the surrounding native species would accelerate its spread in the prairies [3].

Atmospheric CO_2_ concentration has amplified rapidly, from 280 ppm in 1750 at the onset of the Industrial Revolution to greater than 400 ppm currently [66,67]. Increasing CO_2_ concentrations together with increases in other greenhouse gases have resulted in a 0.8 °C rise in mean annual global temperature since 2017 [67]. According to recent projections, the atmospheric CO_2_ concentration will potentially reach up to 800 ppm by the end of the century [68], which would result in a subsequent rise of another 1–3.7 °C in global mean air temperature unless adequate measures are taken to reduce greenhouse gas emissions [66,67,68,69]. Under these elevated atmospheric CO_2_ levels, Kentucky bluegrass, which is a C3 species [70], might be more efficient photosynthetically than C4 grasses. A previous study [70] showed that under a lower level of ambient CO_2_ (less than 300 µL/L), big bluestem (C4 species) had a faster photosynthetic rate than Kentucky bluegrass. Under a higher level of CO_2_, the photosynthetic rate of Kentucky bluegrass increased by 141%, while the big bluestem’s photosynthetic rate remained unchanged. Another study involving Poaceae species reported that although both C3 and C4 grasses exposed to elevated CO_2_ concentrations produced more biomass than grasses exposed to ambient concentrations, C3 grasses produced about 10% more biomass and 27% more tillers compared to C4 grasses [71]. Kentucky bluegrass is already a fast-growing, rhizomatous C3 grass, and increased atmospheric CO_2_ levels could further enhance its productivity.

Climate change has resulted in increased precipitation in the NGP [3,72]. Historical climate data from central North Dakota demonstrated a steady increase in precipitation in the last 130 years [3]. Furthermore, precipitation data from Mandan, North Dakota, showed that the average annual precipitation of 10 years between 1990 and 2000 was approximately 15% greater than that of the preceding 75 years [3,73]. Interestingly, a considerable increase in Kentucky bluegrass spread was reported during the same period [3]. Being a drought-intolerant and hydrophilic species, Kentucky bluegrass tends to invade mesic prairies [3,34,36,74,75]. A previous study indicated a significant positive association between upsurges in the Kentucky bluegrass cover and increasing precipitation in the NGP [71]. In contrast, forbs dominated these areas during the dry periods [76]. Thus, increasing precipitation probably would promote the growth and spread of Kentucky bluegrass in the NGP. Furthermore, a substantial increase in C3 grass production under increased precipitation was observed only under elevated CO_2_ concentration, indicating the possible future dominance of C3 grasses [72,77], including Kentucky bluegrass.

### 4.4. Nitrogen Deposition

**Figure 2:**
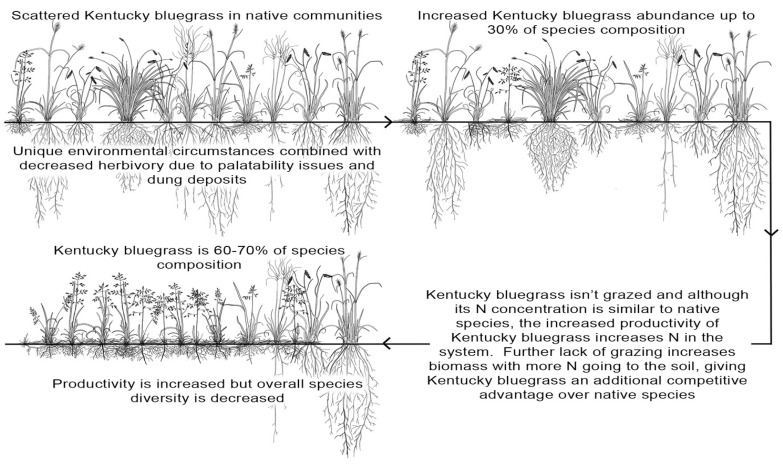
Relationship between Kentucky bluegrass spread and increasing soil nitrogen content. Conceptual figure developed by Robert Pennington, permission to use granted by John Hendrickson and David Toledo, U.S. Department of Agriculture, Agricultural Research Service, Mandan, ND.

Nitrogen is a vital and often limited plant nutrient, but many ecosystems contain greater plant diversity when the available nitrogen is limited [78,79]. Current global estimates suggest that most areas will experience increased atmospheric nitrogen deposition by 2030 [78,80], possibly resulting in a significant loss of global plant biodiversity [43,81]. Natural disturbances, such as fire and grazing, influenced the formation of the mixed-grass prairie of the NGP [6,82]. In the native mixed-grass prairies, the available nitrogen remains sequestered in soil organic matter, and prairie fires reduce the total nitrogen through volatilization and slowing the conversion of organic nitrogen from a labile to a recalcitrant form [6,83]. Moreover, fire negatively affects soil microbial activity, which in turn slows the available nitrogen cycling [83,84]. Most native grass and forbs species have a high carbon:nitrogen ratio, which delays decomposition and reduces available nitrogen required for plant growth [6]. However, a change from a diverse native community to a Kentucky bluegrass-dominated plant community characterized by reduced carbon:nitrogen ratios might make nitrogen more available to plants, eventually altering overall nutrient cycles [85]. In general, prairie systems are vulnerable to small alterations of available nitrogen. Although the added nitrogen increases the overall production, many native species lose their natural ability to compete that they used to have under lower nitrogen circumstances, and the plant diversity declines [6,46,86]. These changes in the available nitrogen levels as a result of changes in community structure, fertilization, and atmospheric nitrogen deposition may result in the faster spread and dominance of Kentucky bluegrass in prairie ecosystems [6,46,86]. The shift of the prairie plant community from native grass and forb dominated to Kentucky bluegrass dominated grassland reduces soil surface fire intensity by altering the fuel properties, including the distribution and moisture, which in turn results in decreased nitrogen volatilization [64,87]. This excess available nitrogen in the ecosystem potentially shifts the competitive advantage to invasive species that are a better fit in a nitrogen-rich environment than the native species [64,86,88]. For example, in a previous study [89], when there was a lack of herbivory because of low palatability and dung deposition, Kentucky bluegrass abundance increased up to 30% of the species composition (Figure 2). After that, grazing was stopped, and expanding Kentucky bluegrass cover increased available nitrogen in the system. The absence of grazing increased Kentucky bluegrass biomass and the available nitrogen in the soil, which enhanced this species’ competitive advantage. Such excess nitrogen levels may also be harmful to mycorrhiza, which favors the growth and establishment of non-mycorrhizal Kentucky bluegrass [64,90].

## 5. Requirement of Efficient Management Strategies

**Figure 3:**
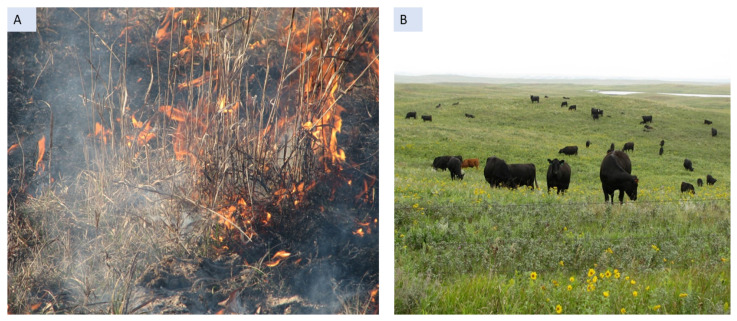
Prescribed seasonal burning (**A**) and season-long grazing (**B**) are the most common management practices used to combat the growth and spread of Kentucky bluegrass in the NGP. Photo (**A**) was taken by Kelly Krabbenhoft and (**B**) by Edward S. DeKeyser.

The NGP consists predominantly of mixed-grass prairies, composed of tallgrass and shortgrass species. As this mixed-grass prairie is situated as an ecotone between the tallgrass and shortgrass prairies, the vegetation is composed of warm and cool-season species from both types of prairies. Thus, these rangelands need both warm and cool-season species management plans [20,25]. Grasslands in NGP historically evolved under disturbances, including fire (both anthropogenic and natural) and bison grazing along with inconsistent climate variability until they were protected by the United States Fish and Wildlife Service (Service) in the mid-1960s [91]. These Service-owned protected prairies were rested to increase undisturbed, dense native cover for the prairie birds almost until 1990. This rest might have encouraged the invasion of some cool-season introduced grasses [91,92]. Application of prescribed fire and grazing increased significantly since 1990s. Disturbance management practices, including prescribed fire, grazing, and a combination of both (i.e., patch burning) are standard management plans for controlling Kentucky bluegrass [20,30] (Figure 3). However, because Kentucky bluegrass shares morphological and physiological similarities with many native cool-season grasses, controlling this non-native grass species with grazing and fire without harming the native species can be difficult [6]. A previous study reported that controlling invasive cool-season Kentucky bluegrass in NGP was particularly difficult when surrounded by native cool-season grasses, especially in the sites where management plans were passive or rested [91]. Moreover, ongoing climate change and increased deposition from atmospheric nitrogen have contributed to rapid nitrogen mineralization [6,78], which would potentially influence, when combined with burning, the invasion and spread of Kentucky bluegrass in the NGP. Together, these practices form a positive feedback mechanism that affects the performance of Kentucky bluegrass in the invaded range. Efficient management strategies that would simultaneously control the spread of invasive species and facilitate the growth and establishment of the native species [93,94,95] are urgently needed.

### 5.1. Modification of Existing Management Practices

Prairie ecosystem management is often complicated by steep challenges. In addition to adopting alternative management practices, land managers also need modifications in their existing plans to sustain ecosystem balances and preserve biodiversity [28,96,97]. Traditional restoration and conservation goals and strategies may require substantial adjustment to contend with the reality of emergent or novel ecosystems [27]. Responses of plants to specific management tools (fire and grazing) often vary with the topographic, edaphic, and climatic gradients. Potential mechanisms behind these variations in plant responses (such as competition) could be proposed as the working hypotheses in adaptive management frameworks [91]. These hypotheses could be used as simple models to be field-tested across a wide range of physiographic regions and climatic gradients, with outcomes informing adjustment of future management approaches to mitigate the spread of the invasive species. Invasive species and the surrounding plant community are variably influenced by the type, timing, intensity, and frequency of anthropogenic disturbances (i.e., burning and grazing) [93,98]. Fire has been successfully used to combat invasive grasses and promote native species. However, limited knowledge exists about the role of burning in controlling invasive species when the invasive plants share similar phenological traits with the surrounding native species [6,99]. A previous study [93] demonstrated that, in the cool-season dominated rangelands, late-growing and dormant season prescribed fires more successfully controlled Kentucky bluegrass than early-season fires in the NGP. As the native species had lower mortality rates than the Kentucky bluegrass during the late-growing season and dormant season fires, native species cover increased significantly. Moreover, under greater fuel loads, both Kentucky bluegrass and other perennial native grasses had greater mortality rates, but Kentucky bluegrass experienced the most damage [93]. Similar to the variations in prescribed burning practices, modification of grazing practices also showed promising results for controlling Kentucky bluegrass invasion in the NGP. Dornbusch et al. [96] showed that although prescribed season-long grazing enhanced Kentucky bluegrass abundance by approximately 20%, alternative grazing methods such as early-season intensive grazing and patch burning maintained species richness in relation to the level seen at the start of the study. Additionally, native species cover was also significantly greater under alternative grazing treatments. Application of herbicide, such as glyphosate, is a popular rangeland management practice [31,32]. In the NGP, the combination of burning and glyphosate application during early spring and late fall when the warm season grasses were dormant showed promising results in combating Kentucky bluegrass while increasing the native grasses [32]. Thus, alteration in traditional practices could be a potential strategy to control the Kentucky bluegrass invasion while improving the overall native species cover.

### 5.2. Competition with Native Species as a Prospective Management Tool

The control and management of Kentucky bluegrass cannot only depend on the traditional management strategies such as fire, grazing, or herbicides—innovative ecological approaches are important to mitigate the invasion of this species. Enhancing the success of native competitors of Kentucky bluegrass could be an essential management strategy for this invasive species [91]. Previous studies suggested that a native or an invasive species’ competitive success is often affected by the available resources [100,101,102]. Several previous studies indicated that increased nitrogen levels favor exotic species over the natives in different ecosystems [103,104,105], yet little information is available on how available soil nitrogen affects competition between the exotic and native perennial grasses [106]. In a greenhouse competition study between the native perennial *Hordeum brachyantherum* and an annual exotic *Lolium multiflora*, an increased level of nitrogen reduced the competitive ability of the native species [107]. Exotic perennial grasses might be more competitive compared with native grasses in a nitrogen-enriched environment [105]. Studies about native grass responses to nitrogen fertilization would clarify whether increased available nitrogen would favor the establishment and spread of Kentucky bluegrass.

Exotic species typically outperform native species under high-resource environments compared to low resource environments [107]. However, exotic species’ performance largely relies on traits of those species and the surrounding native species [108]. According to the limiting similarity hypothesis, species with similar functional traits are better competitors for available resources [109,110]. For example, competition for available water, nutrients, and light during the seedling development stage is the determining factor for species success, and species with similar functional traits such as root structure and resource uptake mechanisms are better competitors [109,111,112]. A previous study found that invasive grasses (Kentucky bluegrass and smooth brome) were equally competitive with a few native grasses, including *Elymus canadensis* (Canada wildrye) and *Pascopyrum smithii* (western wheatgrass), under variable available moisture [113]. This suggests that if certain native species occupy similar ecological niches as Kentucky bluegrass, these native species may be able to outcompete this invasive grass. Often, invasive plants exhibit priority effects whereby they commence growth earlier in the spring than their native neighbors [114]. Priority effects may significantly influence the competition between the invasive and native species and subsequently alter the surrounding plant community’s composition. However, only a few studies have examined the influence of priority effects on the competition between invasive and native species [114,115].

## 6. Outlook

Invasion is a complex and dynamic process that involves several abiotic and biotic factors. Little research has been conducted to assess the underlying mechanisms and feedbacks that favor the establishment and the spread of Kentucky bluegrass [6]. Vital feedback between plants and their soil microbiota can explain why some plant species invade specific ecosystems and how invasion can lead to long-term and irreversible changes in soil and plant communities’ composition and function [116,117,118]. Despite the recent dominance of Kentucky bluegrass in the NGP, little is known about how this invasive grass establishes itself in a new range and how it alters the native soil microbiota.

A Kentucky bluegrass-invaded site will likely not spontaneously be restored to a previous state dominated by native grassland species [49,50]. However, the specific mechanisms and feedbacks aiding the introduction, establishment, and invasion of Kentucky bluegrass in the native rangeland ecosystems remain poorly understood [6]. Exotic species invasions and their impacts on native flora are often associated with nutrient enrichment caused by increased nitrogen pollution or nitrogen fertilization [119,120,121,122]. Although previous studies indicated that reduced native species richness and diversity are associated with Kentucky bluegrass invasion, specific causal mechanisms are still unknown.

In conclusion, additional research focusing on Kentucky bluegrass ecology and the surrounding native plant community and various biotic and abiotic factors is essential in developing efficient management strategies to combat the spread of Kentucky bluegrass in native prairies. As novel ecosystems present unique challenges, considerable time, trial, and evaluation of different prospective management approaches will be required to establish the appropriate adaptive management models. Modifications in existing management plans, such as modifying the type, timing, intensity, and frequency of fire, grazing, or herbicides applications, might help achieve the optimal management strategies for this invasive species. Furthermore, the inclusion of science-based alternative approaches aiming to enhance the competitiveness of the surrounding native grasses, and the role of plant-soil feedback in Kentucky bluegrass invasion, could also be important.

## References

[1] Grant T.A., Shaffer T.L., Flanders B. (2020). Resiliency of native prairies to invasion by Kentucky bluegrass, smooth brome, and woody vegetation. Rangel. Ecol. Manag..

[2] Kobiela B., Quast J., Dixon C., DeKeyser E.S. (2017). Targeting introduced species to improve plant community composition on USFWS-managed prairie remnants. Nat. Areas J..

[3] DeKeyser E.S., Dennhardt L.A., Hendrickson J. (2015). Kentucky bluegrass (Poa pratensis) Invasion in the Northern Great Plains: A story of rapid dominance in an endangered ecosystem. Invasive Plant Sci. Manag..

[4] DeKeyser S., Clambey G., Krabbenhoft K., Ostendorf J. (2009). Are changes in species composition on Central North Dakota Rangelands due to non-use management?. Rangelands.

[5] Samson F., Knopf F. (1994). Prairie Conservation in North America. Bioscience.

[6] Toledo D., Sanderson M., Spaeth K., Hendrickson J., Printz J. (2014). Extent of Kentucky bluegrass and its effect on native plant species diversity and ecosystem Services in the Northern Great Plains of the United States. Invasive Plant Sci. Manag..

[7] DiTomaso J.M. (2000). Invasive weeds in rangelands: Species, impacts, and management. Weed Sci..

[8] Mack R.N. (1981). Invasion of *Bromus tectorum L*. into Western North America: An ecological chronicle. Agro-Ecosystems.

[9] Levine J.M., Vilà M., Antonio C.M.D., Dukes J.S., Grigulis K., Lavorel S. (2003). Mechanisms underlying the impacts of exotic plant invasions. Proc. R. Soc. B Biol. Sci..

[10] Wilcove D.S., Rothstein D., Dubow J., Phillips A., Losos E. (1998). Quantifying threats to imperiled species in the United States. Bioscience.

[11] Samson F.B., Knopf F.L., Ostlie W.R. (2004). Great Plains ecosystems: Past, present, and future. Wildl. Soc. Bull..

[12] Hoekstra J.M., Boucher T.M., Ricketts T.H., Roberts C. (2004). Confronting a biome crisis: Global disparities of habitat loss and protection. Ecol. Lett..

[13] Hendrickson J.R., Sedivec K.K., Toledo D., Printz J. (2019). Challenges facing grasslands in the Northern Great Plains and North Central Region. Rangelands.

[14] Grant T.A., Shaffer T.L., Flanders B. (2020). Patterns of Smooth Brome, Kentucky bluegrass, and shrub invasion in the Northern Great Plains vary with temperature and precipitation. Nat. Areas J..

[15] Pimentel D., McNair S., Janecka J., Wightman J., Simmonds C., O’Connell C., Wong E., Russel L., Zern J., Aquino T. (2001). Economic and environmental threats of alien plant, animal, and microbe invasions. Agric. Ecosyst. Environ..

[16] Fournier A., Penone C., Pennino M.G., Courchamp F. (2019). Predicting future invaders and future invasions. Proc. Natl. Acad. Sci. USA.

[17] Stohlgren T.J., Schnase J.L. (2006). Risk analysis for biological hazards: What we need to know about invasive species. Risk Anal..

[18] Pimentel D., Zuniga R., Morrison D. (2005). Update on the environmental and economic costs associated with alien-invasive species in the United States. Ecol. Econ..

[19] Sohrabi S., Downey P.O., Gherekhloo J., Hassanpour-Bourkheili S. (2020). Testing the Australian post-border Weed Risk Management (WRM) system for invasive plants in Iran. J. Nat. Conserv..

[20] Ellis-Felege S.N., Dixon C.S., Wilson S.D. (2013). Impacts and management of invasive cool-season grasses in the Northern Great Plains: Challenges and opportunities for wildlife. Wildl. Soc. Bull..

[21] McGregor R.L., Barkley T.M., Great Plains Flora Association (1986). Flora of the Great Plains.

[22] Schulte J.R. (2011). The Fire Ecology of Kentucky Bluegrass (*Poa pratensis*). Master’s Thesis.

[23] Huff D.R., Bara J.M. (1993). Determining genetic origins of aberrant progeny from facultative apomictic Kentucky bluegrass using a combination of flow cytometry and silver-stained RAPD markers. Theor. Appl. Genet..

[24] Mack R.N., Simberloff D., Lonsdale W.M., Evans H., Clout M., Bazzaz F.A. (2000). Biotic invasions: Causes, epidemiology, global consequences, and control. Ecol. Appl..

[25] DeKeyser E.S., Meehan M., Clambey G., Krabbenhoft K. (2013). Cool Season invasive grasses in Northern Great Plains natural Areas. Nat. Areas J..

[26] Cully A.C., Cully J.F., Hiebert R.D. (2003). Invasion of exotic plant species in tallgrass prairie fragments. Conserv. Biol..

[27] Estes J.A., Terborgh J., Brashares J.S., Power M.E., Berger J., Bond W.J., Carpenter S.R., Essington T.E., Holt R.D., Jackson J.B.C. (2011). Trophic downgrading of planet earth. Science.

[28] Hobbs R.J., Higgs E., Harris J.A. (2009). Novel ecosystems: Implications for conservation and restoration. Trends Ecol. Evol..

[29] Mckinney M.L., Lockwood J.L. (1999). Taxonomic and ecological enhancement of homogenization. Tree.

[30] Gasch C.K., Toledo D., Kral-O’Brien K., Baldwin C., Bendel C., Fick W., Gerhard L., Harmon J., Hendrickson J., Hovick T. (2020). Kentucky bluegrass invaded rangeland: Ecosystem implications and adaptive management approaches. Rangelands.

[31] Link A., Kobiela B., DeKeyser S., Huffington M. (2017). Effectiveness of burning, herbicide, and seeding toward restoring rangelands in Southeastern North Dakota. Rangel. Ecol. Manag..

[32] Ereth C.B., Hendrickson J.R., Kirby D., DeKeyser E.S., Sedivec K.K., West M.S. (2017). Controlling Kentucky Bluegrass with herbicide and burning is influenced by invasion level. Invasive Plant Sci. Manag..

[33] United States Department of Agriculture, Natural Resources Conservation Service Plants Profile for Poa pratensis (Kentucky bluegrass). https://plants.usda.gov/core/profile?symbol=POPR.

[34] Huff D.R., Casler M., Duncan R. (2003). Kentucky Bluegrass. Turfgrass Biology, Genetics, and Breeding.

[35] Carrier L., Bort S.K. (1916). The history of Kentucky bluegrass and white clover in the United States. Agron. J..

[36] Uchytil R.J. (1993). Poa pratensis. Fire Effects Information System.

[37] Sedivec K., Barker W. (1997). Selected North. Dakota and Minnesota Range Plants EB-69.

[38] Hurto K.A., Turgeon A.J., Spomer L.A. (1980). Physical characteristics of thatch as a turfgrass growing medium 1. Agron. J..

[39] Garrison M.A., Stier J.C. (2010). Cool-Season turfgrass colony and seed survival in a restored prairie. Crop Sci..

[40] Setter C.M., Lym R.G. (2013). Change in leafy Spurge (Euphorbia esula) density and soil Seedbank Composition 10 Years following Release of *Aphthona* spp. Biological Control Agents. Invasive Plant Sci. Manag..

[41] VanEss B.M., Wilson S.D. (2007). Impact and management of crested wheatgrass (*Agropyron cristatum*) in the northern Great Plains. Can. J. Plant Sci..

[42] Pritekel C., Whittemore-Olson A., Snow N., Moore J.C. (2006). Impacts from invasive plant species and their control on the plant community and belowground ecosystem at Rocky Mountain National Park, USA. Appl. Soil Ecol..

[43] Vitousek P.M., Harold M.A., Jane L., Jerry M.M. (1997). Human domination of earth’s ecosystems. Science.

[44] Theoharides K.A., Dukes J.S. (2007). Plant invasion across space and time: Factors affecting nonindigenous species success during four stages of invasion. New Phytol..

[45] Wedin D.A. (1995). Species, Nitrogen, and grassland dynamics: The constraints of stuff. Linking Species & Ecosystems.

[46] Tilman D. (1987). Secondary succession and the pattern of plant dominance along experimental nitrogen gradients. Ecol. Monogr..

[47] Tatina R. (1994). Ordination and description of prairie plant communities from the southern Missouri Coteau in South Dakota. Gt. Plains Res..

[48] Emery S.M., Gross K.L. (2007). Dominant species identity, not community evenness, regulates invasion in experimental grassland plant communities. Ecology.

[49] Wedin D.A., Pastor J. (1993). Nitrogen mineralization dynamics in grass monocultures. Oecologia.

[50] DeAngelis D.L. (1992). Dynamics of Nutrient Cycling and Food Webs.

[51] Sanderson M.A., Johnson H., Liebig M.A., Hendrickson J.R., Duke S.E. (2017). Kentucky bluegrass invasion alters soil carbon and vegetation structure on Northern mixed-Grass prairie of the United States. Invasive Plant Sci. Manag..

[52] Mahaney W.M., Smemo K.A., Gross K.L. (2008). Impacts of C4 grass introductions on soil carbon and nitrogen cycling in C3-dominated successional systems. Oecologia.

[53] Piper C.L., Lamb E.G., Siciliano S.D. (2015). Smooth brome changes gross soil nitrogen cycling processes during invasion of a rough fescue grassland. Plant. Ecol..

[54] Spaeth K.E., Pierson F.B., Weltz M.A., Awang J.B. (1996). Gradient analysis of infiltration and environmental variables as related to rangeland vegetation. Biomass.

[55] Lockwood J.L., Cassey P., Blackburn T. (2005). The role of propagule pressure in explaining species invasions. Trends Ecol. Evol..

[56] Duncan R.P., Blackburn T.M., Rossinelli S., Bacher S. (2014). Quantifying invasion risk: The relationship between establishment probability and founding population size. Methods Ecol. Evol..

[57] Dawson W., Burslem D.F.R.P., Hulme P.E. (2009). Factors explaining alien plant invasion success in a tropical ecosystem differ at each stage of invasion. J. Ecol..

[58] Kolar C.S., Lodge D.M. (2001). Progress in invasion biology: Predicting invaders. Trends Ecol. Evol..

[59] Lockwood J.L., Cassey P., Blackburn T.M. (2009). The more you introduce the more you get: The role of colonization pressure and propagule pressure in invasion ecology. Divers. Distrib..

[60] Barney J.N. (2006). North American history of two invasive plant species: Phytogeographic distribution, dispersal vectors, and multiple introductions. Biol. Invasions.

[61] Hall C.R., Hodges A.W., Haydu J.J. (2006). The economic impact of the green industry in the United States. HortTechnology.

[62] Honig J.A., Bonos S.A., Meyer W.A. (2010). Isolation and characterization of 88 polymorphic microsatellite markers in Kentucky bluegrass (*Poa pratensis* L.). HortScience.

[63] Dennhardt L.A., DeKeyser E.S., Tennefos S.A., Travers S.E. (2016). There is no evidence of geographical patterning among invasive Kentucky bluegrass (*Poa pratensis*) populations in the Northern Great Plains. Weed Sci..

[64] Printz J.L., Hendrickson J.R. (2015). Impacts of Kentucky bluegrass invasion (*Poa pratensis* L.) on ecological processes in the Northern Great Plains. Rangelands.

[65] Badh A., Akyuz A., Vocke G., Mullins B. (2009). Impact of climate change on the growing seasons in select cities of North Dakota, United States of America. Int. J. Clim. Chang. Impacts Responses.

[66] Thirkell T.J., Pastok D., Field K.J. (2019). Carbon for nutrient exchange between arbuscular mycorrhizal fungi and wheat varies according to cultivar and changes in atmospheric carbon dioxide concentration. Glob. Chang. Biol..

[67] Dusenge M.E., Duarte A.G., Way D.A. (2018). Plant carbon metabolism and climate change: Elevated CO2 and temperature impacts on photosynthesis, photorespiration and respiration. New Phytol..

[68] Meinshausen M., Smith S.J., Calvin K., Daniel J.S., Kainuma M.L.T., Lamarque J.F., Matsumoto K., Montzka S.A., Raper S.C.B., Riahi K. (2011). The RCP greenhouse gas concentrations and their extensions from 1765 to 2300. Clim. Chang..

[69] Ciais P., Sabine C., Bala G., Stocker T.F., Qin D., Plattner G.K. (2013). Carbon and other biogeochemical cycles. Climate Change 2013: The Physical Science Basis. Contribution of Working Group I to the Fifth Assessment Report of the Intergovernmental Panel on Climate Change.

[70] He H., Kirkham M.B., Lawlor D.J., Kanemasu E.T. (1992). Photosynthesis and water relations of big bluestem (C4) and Kentucky bluegrass (C3 ) under high concentration carbon dioxide. Trans. Kans. Acad. Sci..

[71] Wand S.J.E., Midgley G.F., Jones M.H., Curtis P.S. (1999). Responses of wild C4 and C3 grass (*Poaceae*) species to elevated atmospheric CO2 concentration: A meta-analytic test of current theories and perceptions. Glob. Chang. Biol..

[72] Dennhardt L.A. (2016). Evidence of Climate Niche Creation in the Northern Great Plains: The History of Invasion, Population Genetics, Competitive Effect, and Long-Term Trends of Poa Pratensis L..

[73] Regional Climate Centers–National Oceanic and Atmospheric Administration (2014). High Plains Regional Climate Center. http://www.hprcc.unl.edu/data/historical/index.php?state5nd&action5se-lect_state&submit5Select+State.

[74] Stevens O.A. (1953). Handbook of North Dakota Plants.

[75] Lowe E. (1858). A Natural History of British Grasses.

[76] Clark J.S., Grimm E.C., Donovan J.J., Fritz S.C., Engstrom D.R., Almendinger J.E. (2002). Drought cycles and landscape responses to past aridity on prairies of the Northern Great Plains, USA. Ecology.

[77] Nie D., Kirkham M.B., Ballou L.K., Lawlor D.J., Kanemasu E.T. (1992). Changes in prairie vegetation under elevated carbon dioxide levels and two soil moisture regimes. J. Veg. Sci..

[78] Bobbink R., Hicks K., Galloway J., Spranger T., Alkemade R., Ashmore M., Bustamante M., Cinderby S., Davidson E., Dentener F. (2010). Global assessment of nitrogen deposition effects on terrestrial plant diversity: A synthesis. Ecol. Appl..

[79] Bobbink R., Hornung M., Roelofs J.G.M. (1998). The effects of air-borne nitrogen pollutants on species diversity in natural and semi-natural European vegetation. J. Ecol..

[80] Dentener F., Drevet J., Lamarque J.F., Bey I., Eickhout B., Fiore A.M., Hauglustaine D., Horowitz L.W., Krol M., Kulshrestha U.C. (2006). Nitrogen and sulfur deposition on regional and global scales: A multimodel evaluation. Glob. Biogeochem. Cycles.

[81] Phoenix G.K., Hicks W.K., Cinderby S., Kuylenstierna J.C.I., Stock W.D., Dentener F.J., Giller K.E., Austin A.T., Lefroy R.D.B., Gimeno B.S. (2006). Atmospheric nitrogen deposition in world biodiversity hotspots: The need for a greater global perspective in assessing N deposition impacts. Glob. Chang. Biol..

[82] Bowman D.M.J.S., Balch J.K., Artaxo P., Bond W.J., Carlson J.M., Cochrane M.A., D’Antonio C.M., DeFries R.S., Doyle J.C., Harrison S.P. (2009). Fire in the Earth System. Science.

[83] Goergen E.M., Chambers J.C. (2009). Influence of a native legume on soil N and plant response following prescribed fire in sagebrush steppe. Int. J. Wildland Fire.

[84] Certini G. (2005). Effects of fire on properties of forest soils: A review. Oecologia.

[85] Hendrickson J.R., Wienhold B.J., Berdahl J.D. (2001). Decomposition Rates of Native and Improved Cultivars of Grasses in the Northern Great Plains. Arid. Land Res. Manag..

[86] Wedin D.A., Tilman D. (1990). Species effects on nitrogen cycling: A test with perennial grasses. Oecologia.

[87] Brooks M.L., D’Antonio C.M., Richardson D.M., Grace J.B., Keeley J.E., DiTomaso J.M., Hobbs R.J., Pellant M., Pyke D.A. (2004). Effects of Invasive Alien Plants on Fire Regimes. Bioscience.

[88] Bradley B.A., Htonw R.A.H.G., Mustard J.F., Hamburg S.P. (2006). Invasive grass reduces aboveground carbon stocks in shrublands of the Western US. Glob. Chang. Biol..

[89] Toledo D., Hendrickson J.R. (2020). Relation of Soil Nitrogen Based on the Extent Kentucky Bluegrass (*Poa pratensis* L.) Invasion in the Northern Great Plains. Personal Communication.

[90] Grygiel C.E., Norland J.E., Biondini M.E. (2010). Can carbon and phosphorous amendments increase native forbs in a restoration process? A case study in the Northern tall-grass rairie (U.S.A.). Restor. Ecol..

[91] Grant T.A., Flanders-Wanner B., Shaffer T.L., Murphy R.K., Knutsen G.A. (2009). An emerging crisis across Northern Prairie Refuges: Prevalence of invasive plants and a plan for adaptive management. Ecol. Restor..

[92] Murphy R.K., Grant T.A. (2005). Land management history and floristics in mixed-grass prairie, North Dakota, USA. Nat. Areas J..

[93] Kral K., Limb R., Ganguli A., Hovick T., Sedivec K. (2018). Seasonal prescribed fire variation decreases inhibitory ability of Poa pratensis L. and promotes native plant diversity. J. Environ. Manag..

[94] Mangla S., Sheley R.L., James J.J., Radosevich S.R. (2011). Intra and interspecific competition among invasive and native species during early stages of plant growth. Plant. Ecol..

[95] Brown C.S., Anderson V.J., Claassen V.P., Stannard M.E., Wilson L.M., Atkinson S.Y., Bromberg J.E., Grant T.A., Munis M.D. (2008). Restoration ecology and invasive plants in the semiarid West. Invasive Plant Sci. Manag..

[96] Dornbusch M.J., Limb R.F., Gasch C.K. (2018). Facilitation of an exotic grass through nitrogen enrichment by an exotic legume. Rangel. Ecol. Manag..

[97] Kirkman L.K., Barnett A., Williams B.W., Hiers J.K., Pokswinski S.M., Mitchell R.J. (2013). A dynamic reference model: A framework for assessing biodiversity restoration goals in a fire-dependent ecosystem. Ecol. Appl..

[98] Jauni M., Gripenberg S., Ramula S. (2015). Non-native plant species benefit from disturbance: A meta-analysis. Oikos.

[99] Simmons M.T., Windhager S., Power P., Lott J., Lyons R.K., Schwope C. (2007). Selective and non-selective control of invasive plants: The short-term effects of growing-season prescribed fire, herbicide, and mowing in two texas prairies. Restor. Ecol..

[100] Lang M., Hanslin H.M., Kollmann J., Wagner T. (2017). Suppression of an invasive legume by a native grass: High impact of priority effects. Basic Appl. Ecol..

[101] Leskovšek R., Eler K., Batič F., Simončič A. (2012). The influence of nitrogen, water and competition on the vegetative and reproductive growth of common ragweed (*Ambrosia artemisiifolia* L.). Plant. Ecol..

[102] Hwang B.C., Lauenroth W.K. (2007). Effect of nitrogen, water and neighbor density on the growth of Hesperis matronalis and two native perennials. Biol. Invasions.

[103] Huenneke L.F., Hamburg S.P., Koide R., Mooney H.A., Vitousek P.M. (1990). Effects of soil resources on plant invasion and community structure in Californian Serpentine Grassland. Ecology.

[104] Limpens J., Berendse F., Klees H. (2003). N deposition affects N availability in interstitial water, growth of Sphagnum and invasion of vascular plants in bog vegetation. New Phytol..

[105] Lowe P.N., Lauenroth W.K., Burke I.C. (2003). Effects of nitrogen availability on competition between *Bromus tectorum* and *Bouteloua gracilis*. Plant. Ecol..

[106] Thomsen M.A., Corbin J.D., D’Antonio C.M. (2006). The effect of soil nitrogen on competition between native and exotic perennial grasses from northern coastal California. Plant. Ecol..

[107] Kolb A., Alpert P. (2003). Effects of nitrogen and salinity on growth and competition between a native grass and an invasive congener. Biol. Invasions.

[108] Daehler C.C. (2003). Performance comparisons of co-occurring native and alien invasive plants: Implications for conservation and restoration. Annu. Rev. Ecol. Evol. Syst..

[109] Fagúndez J., Lema M. (2019). A competition experiment of an invasive alien grass and two native species: Are functionally similar species better competitors?. Biol. Invasions.

[110] Funk J.L., Cleland E.E., Suding K.N., Zavaleta E.S. (2008). Restoration through reassembly: Plant traits and invasion resistance. Trends Ecol. Evol..

[111] Yannelli F.A., Koch C., Jeschke J.M., Kollmann J. (2017). Limiting similarity and Darwin’s naturalization hypothesis: Understanding the drivers of biotic resistance against invasive plant species. Oecologia.

[112] Goldberg D.E., Landa K. (1991). Competitive effect and response: Hierarchies and correlated traits in the early stages of competition. J. Ecol..

[113] Stephens M. (2017). Phenotypic Plasticity of Native and Invasive Cool-Season Grasses in Response to Frequency of Moisture Availability.

[114] Dickson T.L., Hopwood J.L., Wilsey B.J. (2012). Do priority effects benefit invasive plants more than native plants? An experiment with six grassland species. Biol. Invasions.

[115] Abraham J.K., Corbin J.D., D’Antonio C.M. (2008). California native and exotic perennial grasses differ in their response to soil nitrogen, exotic annual grass density, and order of emergence. Herbaceous Plant Ecol..

[116] Wurst S., Ohgushi T. (2015). Do plant and soil mediated legacy effects impact future biotic interactions?. Funct. Ecol..

[117] Bever J.D., Dickie I.A., Facelli E., Facelli J.M., Klironomos J., Moora M., Rillig M.C., Stock W.D., Tibbett M., Zobel M. (2010). Rooting theories of plant community ecology in microbial interactions. Trends Ecol. Evol..

[118] Bever J.D. (2003). Soil community feedback and the coexistence of competitors: Conceptual frameworks and empirical tests. New Phytol..

[119] Driscoll D.A., Strong C. (2017). Covariation of soil nutrients drives occurrence of exotic and native plant species. J. Appl. Ecol..

[120] Borer E.T., Seabloom E.W., Gruner D.S., Harpole W.S., Hillebrand H., Lind E.M., Adler P.B., Alberti J., Anderson T.M., Bakker J.D. (2014). Herbivores and nutrients control grassland plant diversity via light limitation. Nat. Cell Biol..

[121] Lai H.R., Mayfield M.M., Gay-Des-Combes J.M., Spiegelberger T., Dwyer J.M. (2015). Distinct invasion strategies operating within a natural annual plant system. Ecol. Lett..

[122] Wassen M.J., Venterink H.O., Lapshina E.D., Tanneberger F. (2005). Endangered plants persist under phosphorus limitation. Nat. Cell Biol..

